# Inter-Rater Reliability between Structured and Non-Structured Interviews Is Fair in Schizophrenia and Bipolar Disorders—A Systematic Review and Meta-Analysis

**DOI:** 10.3390/diagnostics13030526

**Published:** 2023-01-31

**Authors:** Hélio Rocha Neto, Ana Lúcia R. Moreira, Lucas Hosken, Joshua A. Langfus, Maria Tavares Cavalcanti, Eric Arden Youngstrom, Diogo Telles-Correia

**Affiliations:** 1 Medical Faculty, Lisbon University, 1649-028 Lisbon, Portugal; 2 Programa de Pós Graduação em Psiquiatria e Saúde Mental—PROPSAM, Instituto de Psiquiatria, Universidade Federal do Rio de Janeiro—UFRJ, Rio de Janeiro 22290140, RJ, Brazil; 3 Cork Kerry Community Healthcare, T12YE02 Cork, Ireland; 4 Medical Psychology Sector, University Hospital Clementino Fraga Filho, HUCFF/UFRJ, Rio de Janeiro 21941913, RJ, Brazil; 5 Clinical Psychology Program, Department of Psychology and Neuroscience, University of North Carolina at Chapel Hill, Chapel Hill, NC 27599-3270, USA; 6 Medicine Faculty from Centro de Ciências da Saúde—Universidade Federal do Rio de Janeiro—UFRJ, Rio de Janeiro 21941902, RJ, Brazil; 7 Helping Give Away Psychological Science, Chapel Hill, NC 27514, USA; 8 Clinica Universitária de Psiquiatria e Psicologia Médica, Faculdade de Medicina, Universidade de Lisboa, 1649-035 Lisbon, Portugal

**Keywords:** standard diagnostic interview, meta-analysis, schizophrenia, bipolar affective disorder, reliability and validity

## Abstract

We aimed to find agreement between diagnoses obtained through standardized (SDI) and non-standardized diagnostic interviews (NSDI) for schizophrenia and Bipolar Affective Disorder (BD). Methods: A systematic review with meta-analysis was conducted. Publications from 2007 to 2020 comparing SDI and NSDI diagnoses in adults without neurological disorders were screened in MEDLINE, ISI Web of Science, and SCOPUS, following PROSPERO registration CRD42020187157, PRISMA guidelines, and quality assessment using QUADAS–2. Results: From 54231 entries, 22 studies were analyzed, and 13 were included in the final meta-analysis of kappa agreement using a mixed-effects meta-regression model. A mean kappa of 0.41 (Fair agreement, 95% CI: 0.34 to 0.47) but high heterogeneity (*Î*^2^ = 92%) were calculated. Gender, mean age, NSDI setting (Inpatient vs. Outpatient; University vs. Non-university), and SDI informant (Self vs. Professional) were tested as predictors in meta-regression. Only SDI informant was relevant for the explanatory model, leaving 79% unexplained heterogeneity. Egger’s test did not indicate significant bias, and QUADAS–2 resulted in “average” data quality. Conclusions: Most studies using SDIs do not report the original sample size, only the SDI-diagnosed patients. Kappa comparison resulted in high heterogeneity, which may reflect the influence of non-systematic bias in diagnostic processes. Although results were highly heterogeneous, we measured a fair agreement kappa between SDI and NSDI, implying clinicians might operate in scenarios not equivalent to psychiatry trials, where samples are filtered, and there may be more emphasis on maintaining reliability. The present study received no funding.

## 1. Introduction

Low diagnostic reliability threatens the validity of both research and practice in psychiatry [1,2]. Accurate diagnosis forms the bedrock of treatment selection and management of comorbidities, and the lack of a reliable diagnostic process can contribute to variability in outcomes, despite the availability of efficacious treatments. Nevertheless, diagnosing mental disorders poses a serious challenge, in part because of a lack of identifiable and specific biomarkers, leaving clinicians to rely on the evaluation of subjective characteristics susceptible to interpretation and potential bias [3,4,5].

The “operational revolution” popularized the definition of mental disorders using “operational criteria” comprising checklists of signs and symptoms [6]. Such definitions were considered “atheoretical” and thought to reduce the role of clinical judgment or interpretation, which may be tied to a particular conceptual model [6]. A standard diagnostic interview (SDI) is one way to evaluate whether a patient meets the operational definition of a disorder. The companion SDI for the Diagnostic and Statistical Manual (DSM) [7] is the Structured Clinical Interview for DSM (SCID) [8] which has become the prevailing standard for psychiatry research around the world [9].

The move toward operational diagnostic criteria and the use of SDIs aimed to solve the problem of unreliability in psychiatric diagnosis. Despite the increased reliability of SDIs, in practice clinicians often use non-standard diagnostic interviews (NSDI) [10,11]. These may be unstructured, impressionistic, guided by experience and intuition, and prototype-based diagnostic processes and their use can contribute to a gap between research evidence, which typically informs the construction of SDIs, and clinical practice. Nevertheless, NSDIs have some benefits. Evidence-Based Medicine (EBM) is described as the interaction of three areas of knowledge: clinical experience and expertise, patient values and expectations, and the best external evidence [12]. NSDIs can address the complexities that arise in specific cases but sacrifice the first two in favor of the third. Thus, the use of SDIs can err in the opposite direction, causing a tension between “rigor” and “relevance” [13]. Importantly, clinicians can still operationalize standardized diagnostic criteria without using an SDI.

One question is whether clinicians’ diagnoses using NSDIs are less accurate compared to those made with SDIs. Since SDIs are currently considered the gold standard for diagnosis (particularly with a consensus review process), a more tractable question involves examining the agreement between the two approaches. If SDIs and NSDIs disagree, then typical psychiatric practice is at best less accurate and may be subject to systematic biases. Furthermore, if NSDIs do not reproduce the results of SDIs, evidence-based interventions, such as medications, psychotherapies, or alternative treatments, tested in trials with SDIs, are less likely to work as expected for patients diagnosed via NSDIs.

The disjuncture between research and practice may contribute to the shrinkage in treatment effect sizes moving from efficacy to effectiveness designs. Furthermore, NSDIs are subject to local and regional variations in practice. The Dartmouth Medical Atlas Project has found this at every level of analysis within the USA—national, regional, state, and local municipalities—and across every medical specialty examined [14,15]. Current “big data” projects, using statistical and machine-learning models, hinge on the accuracy of NSDIs as they mine medical records and claims data. If NSDIs are fundamentally prone to systematic biases, then these sophisticated models will be trained using unreliable targets and unable to generalize across regions or settings [16]. If they are not, we may consider that the use of SDIs in the research setting is dispensable, and studies using clinical NSDIs only are feasible. This further highlights the importance of understanding the level of agreement between SDIs and NSDIs for diagnostic decision-making.

Little previous work has examined agreement between SDI and NSDI diagnoses. A previous meta-analysis [9] found low agreement between SDI and NSDI diagnoses in children and adolescents. Later, Jensen-Doss [17] found an equivalent result comparing K-SADS and NSDIs, but again in a child and adolescent population. Rettew’s work [9] is the latest review to address this question and is now 15 years old, without adult population evaluation. Thus, in order to update these findings and fill in the gap in adult psychiatry, the current work presents a systematic review of the reliability between SDI and NSDI diagnoses in schizophrenia and Bipolar Affective Disorder (BD) patients, followed by a meta-analysis using kappa agreement as the effect size.

We focused on schizophrenia and BD diagnosis as index disorders, as their diagnostic constructs seem valid and persistent across the world, beyond cultural barriers [18,19,20,21]. This reduces the likelihood that our kappa estimates will be influenced by disagreement about the construct rather than differences between SDIs and NSDIs. As a result, our estimate here may be interpreted as near the upper limit of agreement, with other disorders showing lower overall agreement due to differences in conceptualization.

## 2. Materials and Methods

This review examined studies comparing diagnostic accuracy of SDIs and NSDIs, searching for each SDI by name and acronym. SDIs targeting both schizophrenia and BD (as is the case of SCID [8]) or just one of these diagnoses (as in the Mood Disorder Questionnaire; MDQ [22]) were then selected to build the search string. We initially sought to include the “missing gold standard” or Longitudinal, Expert, All Data (LEAD) approach [23,24]. However, use of “LEAD” in searches yielded few results. Therefore, the following SDIs were included: Composite International Diagnostic Interview—CIDI [25], Diagnostic Interview Schedule—DIS [26], Mini International Neuropsychiatric Interview—MINI [27], Schedules for Clinical Assessment in Neuropsychiatry—SCAN [28], Structured Clinical Interview for DSM—SCID [8], Standard for Clinicians Interview in Psychiatry—SCIP [29], Schedule for Affective Disorders—SADS [30], Diagnostic Interview for Genetic Disorders—DIGD [31], Bipolar Spectrum Diagnostic Scale—BSDS [32], General Behavior Inventory—GBI [33], Mood Disorder Questionnaire—MDQ [22], The Comprehensive Assessment of Symptoms and History—CASH [34]. As a generic reference for SDIs, we also included the term “standard diagnostic interview—SDI”.

We conducted the search in MEDLINE, SCOPUS and ISI Web of Science databases. We restricted the year of publication to 2007 and beyond, since the Rettew et al. meta-analysis had collected data until that year. We augmented the search to include papers published in Portuguese and Spanish, in addition to English, though all articles recovered had an English version. The search string was built using both SDI acronyms and full length in title, abstract, subject and keywords, adapting Boolean operators for each database.

Beyond time span and language, inclusion criteria focused on original articles and reviews as publication type, and clinical trials, meta-analyses, randomized controlled trials, reviews and systematic reviews in research type. There are some reasons for including papers other than original diagnostic studies: Firstly, the number of studies that make a direct comparison between SDI and NSDI were surprisingly low; secondly, it is expected for clinical trials to recruit their patients with existing NSDI diagnoses, then to administer an SDI, and then extract their validated sample, which could give us more data than original diagnostic studies only; thirdly, we hoped to harvest references not included in MEDLINE, SCOPUS and ISI Web of Science through other reviews and meta-analyses. Table 1 details the inclusion and exclusion criteria. For quality assessment, we used the “Standards for Reporting of Diagnostic Accuracy Studies” (STARD) [35] criteria and applied the Quality Assessment of Diagnostic Accuracy Study (QUADAS–2) [36] tool. An “extraction tool” was built to get the information desired from each paper (described later).

### Rater Training and Reliability

Two authors (HGRN and LH) trained to use STARD, QUADAS–2, and the extraction tool in a dummy sample and then independently screened and selected references based on the instrument. Training was done in blocks of 10 papers, with the *a priori* protocol entailing a minimum of 3 training blocks and additional training until a kappa of 0.8 was achieved. After the third trial, inter-coder kappa was 0.81 (“Almost Perfect”; CI 0.69–0.93; *p* < 0.001) and article coding proceeded.

For the meta-analysis explanatory model, 10 variables were extracted: Number of subjects in each sample (*N*), female participants ratio, mean sample age in years, SDI, SDI informant (self vs. professional), informant profession, sample diagnosis, research setting (university vs. non-university), clinical setting (Inpatient vs. Outpatient), and country (later converted in Life Expectancy Index—LEI, using WHO database data, matching country data by publication year [37], as it seemed a better way to measure health system strength than countries name alone). The 2 coders also applied STARD and QUADAS–2 independently. Differences were reviewed directly in the reference or, whenever possible, contacting their authors to resolve any conflicts.

This review protocol was registered in PROSPERO under the registration number CRD42020187157 on the 19 May 2020, before reference extraction. The 3 databases were accessed on the 10 June 2020. This study and report have been designed and written following PRISMA [38] orientation (PRISMA checklist appended).

Agreement (kappa) of SDI vs. NSDI diagnoses was directly extracted from papers where they were already reported or calculated when paper offered enough information or their authors provided it after direct request by email. For the meta-analysis, we followed Jansen’s approach [39]. A power analysis using the *metapower* package v0.2.2^40^ found that an effect size of 0.4 (fair agreement, and roughly the median in the DSM—5 field trials) [40] was detectable at a level of 99.8%, with a median sample size (*N* ~ 114), and 13 studies using a random effects model and high heterogeneity (e.g., *I*^2^ ~ 0.9). Power would have been >86% to detect differences of *k* = 0.4 vs. 0.2 under moderate heterogeneity (*I*^2^ ~ 0.5), though it dropped to 28% under conditions of high heterogeneity for random effects model testing moderators.

Once coded, kappas were pooled, and 95% CI was calculated using a random effects model. After pooled kappa calculation, mixed model meta-regression probed the heterogeneity (*Î*^2^). Statistics were conducted using the *metafor* [41] and *metapower* [42] package for R statistical software (v4.1.2; R Core Team, Vienna, Austria), which also provided the funnel and forest plots.

## 3. Results

**Figure 1:**
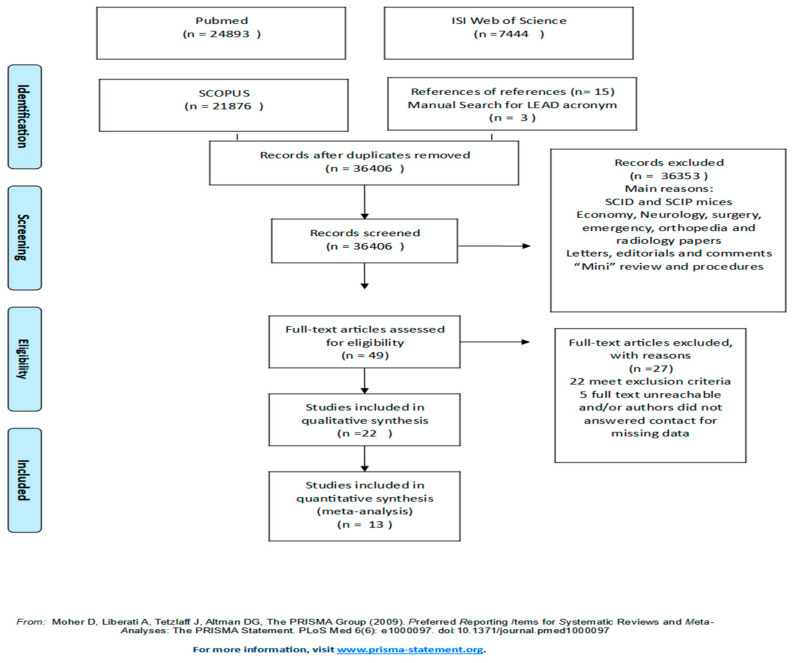
Screening, selection, inclusion and exclusion workflow.

Our search protocol captured 54,231 initial entries. Further applications of inclusion/exclusion criteria, deletion of duplicates and unrelated references resulted in 49 references retained for eligibility assessment. A final list of 13 papers were coded for analysis, providing 15 kappas. Figure 1 presents the flow diagram from search to final inclusion.

SCID was the most reported SDI (*n* = 3872) (based on full length, to avoid cross references with other acronyms), followed by CIDI (*n* = 2662) and MINI (*n* = 2420). DIGD was not found in any reference, and CASH was used in a single report (see Table 2 for details). Almost all years had at least 1 reference in the final list, but only 5 SDIs were represented (SCID, MINI, CIDI, MDQ and BSDS). Table 3 presents the final list of included sources with author, publication year, and diagnosis’ details.

**Figure 2:**
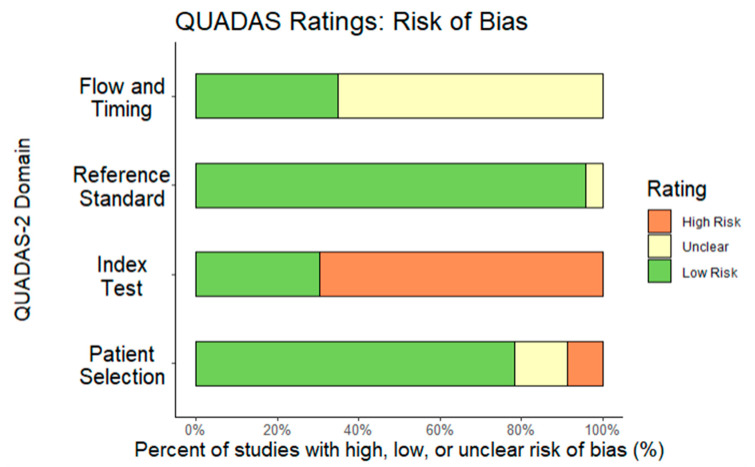
QUADAS Risk of Bias report.

**Figure 3:**
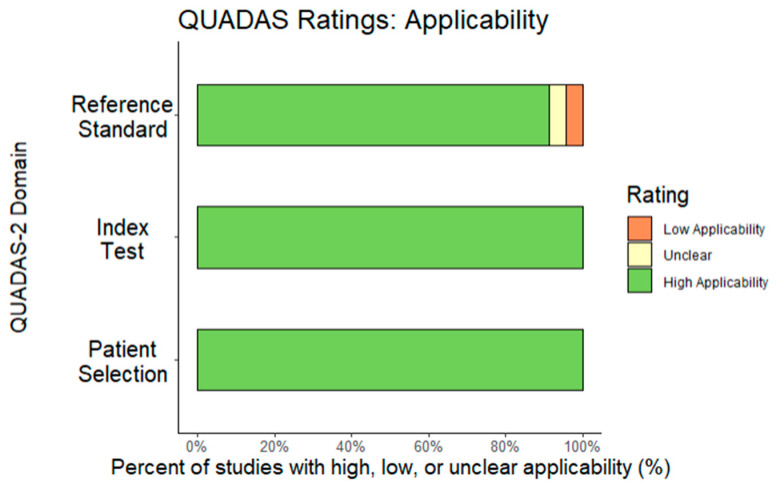
QUADAS Applicability report.

References were of “average” quality based on QUADAS–2 scores. The most common issue was that subjects were usually recruited from settings dedicated to a specific disease or to similar diagnostic spectra (e.g., schizophrenia spectrum) when performing reliability calculations. In two studies, it was not possible to check patient selection bias [51,57], and a third may have excluded patients with previous mood-related psychotic symptoms [62]. In Suresh et al. [53], it was not clear if clinicians knew SDI results (i.e., failure of masking), but that was not an issue for all other references. Whenever a gross disruption in case flow and timing of diagnoses was identified, the reference was excluded (*k* = 1), but in the final sample, only eight studies explicitly reported the interval between SDI and NSDI diagnosis, resulting in most studies receiving an “unknown” classification. Most studies used methodologies considered equivalent to usual clinical settings, except for Nordgaard et al. [47], where the reference standard was a diagnostic consensus among two highly trained researchers in diagnostic interviews. Figure 2 and Figure 3 report the full QUADAS–2 coding.

**Figure 4:**
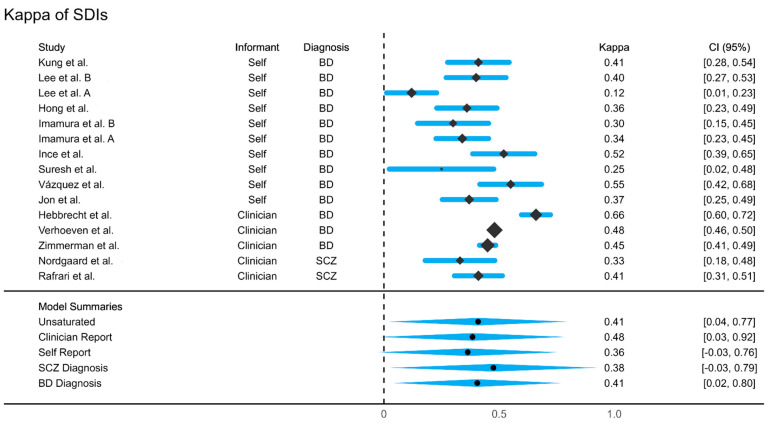
Forest Plot. Refs. [45,47,49,50,51,53,55,56,57,60,61,62,63].

**Figure 5:**
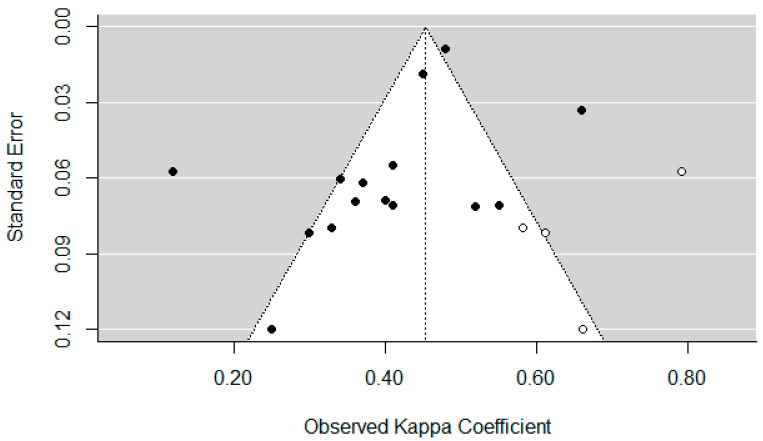
Trim and fill funnel plot. White dots indicate implied missing studies.

Of the final analyzed entries, 15 results were included for meta-analysis. These studies reported kappas ranging from 0.12 to 0.66. The trim-and-fill funnel plot (Figure 4) indicated that if there was bias, it would have been due to unpublished studies having a small sample size and high kappas (e.g., three implied studies in that region of the plot). Egger’s test indicated no significant bias. The weighted mean kappa was 0.41 (Fair agreement, 95% CI: 0.34 to 0.47), however, with a high heterogeneity (*Î*^2^ = 92%) (Figure 5).

An augmented meta-regression model tested female percentage, publication year, NSDI setting (inpatient vs. outpatient; university vs. non-university) and SDI interview (CIDI, MDQ, MINI, BSDS, SCID) as potential moderators. The model accounted for *R*^2^ = 10.1% of variance in kappas, *Q_model_* (8 *df*) = 9.20, *n.s.*, leaving 79% unexplained heterogeneity, *Q_error_* (6 *df*) = 38.31, *p* < 0.00005. An alternate model collapsing SDI interview into a format (self-administered vs. interview) performed similarly: *R*^2^ = 18.8%, *Q_model_* (5 *df*) = 7.50, *n.s.*, leaving 79% unexplained heterogeneity, *Q_error_* (9 *df*) = 54.46, *p* < 0.00005.

## 4. Discussion

The goal of the present study was to meta-analyze agreement between diagnoses based on SDIs versus NSDIs in patients with BD and schizophrenia. The average agreement between the two methods was “fair” based on a literature of “average” reporting quality. High heterogeneity persisted, even after exploring a variety of potential predictors using mixed meta-regressions.

The type of information obtained with SDIs versus NSDIs, as well as clinicians’ use of diagnostic prototypes instead of standardized criteria, may be reasons for the low agreement. However, clinicians’ prototype-based approach usually match ICD or DSM criteria, even with NSDIs as information-gathering procedure [11]. NSDIs allow clinicians’ use of clinical judgment to uncover relevant information not probed in a SDI [64,65]; however, this can also incur biases to jeopardize the evidence-gathering process. Thus, the lack of agreement may be due to different information being uncovered with the use of SDIs versus NSDIs, even with clinicians applying standardized criteria. If SDIs and NSDIs result in different diagnoses, despite the use of operational criteria for the disorders themselves, then research in psychiatry works with diagnostic models that do not represent clinical practice and vice versa.

### 4.1. Assessing Model Heterogeneity

None of the variables examined as potential moderators significantly reduced heterogeneity in kappa estimates. Previous studies suggested that patients give more information and are more reliable in their statements on self-reporting instruments compared to clinician-guided interviews, particularly about sensitive or stigmatized topics [66]. However, self-administered interviews may lead to failure to accurately report symptoms due to difficulty in comprehending technical language [67]. Additionally, both mania and psychosis can involve a lack of insight into one’s mental state or behavior. It is possible that patients misunderstood questions, reported more information than requested by doctors or did not classify certain signs and symptoms in the same way a clinician would [68].

Considering the other explanatory variables, we anticipated that a semi-structured format would be more sensitive and specific than a fully structured SDI. However, the retrieved studies largely did not report which format was used, with the exception of Nordgaard et al. [63], who raised this hypothesis. Thus, the effect of format (structured vs. semi-structured) could not be tested as a heterogeneity explanation.

We also expected that strong public health systems would be associated with better practices, more professional training, and the adoption of quality protocols. Using Life Expectancy Index (LEI) as a proxy for health system quality, we tested whether it would explain further reliability between SDI and NSDI; however, it had no impact on our explanatory model.

The setting where NSDI was performed was also expected to be a predictor of heterogeneity. University settings might be more adherent to diagnostic protocols and have clinicians that are up to date regarding diagnostic protocols compared to non-university services. Furthermore, we expected to see a difference between inpatient and outpatient clinics due to the number of assessments and intensity of behavior observation. However, none of these factors were significant in the explanatory model.

### 4.2. Limits for Systematic Review of Agreement Studies

This review was limited by the number of studies that reported adequate information for coding, which represented <1% of the citations captured in the pre-registered search strategy. Furthermore, although most studies showed a QUADAS–2 rating of “adequate” quality (Figure 2), we encountered challenges due to inadequate reporting, including difficulty extracting information about potential moderators, as well as an extremely low yield of usable studies compared to initial search results.

There were several common weaknesses in the reporting of results that resulted in the exclusion of potentially interesting predictors of agreement. Since SDIs are the dominant standard for research in psychiatry, we expected studies to report agreement statistics of NSDIs vs. SDIs as part of the study (e.g., patients initially diagnosed with schizophrenia using NSDI then recruited for research and tested with a SDI to confirm diagnosis). Unfortunately, very few papers reported the initial number of tested subjects and most reported only SDI-positive recruited participants. This makes it impossible to estimate the base rate, the kappa, and other statistics needed to assess agreement between SDIs and NSDIs [69].

Another challenge in reviewing the literature was that studies often used a specific module of SDI instead of the whole instrument. Both DSM and ICD have exclusion criteria for disorders that should render impossible at least some types of comorbidity (such as schizophrenia and BD). Triage tools developed for a single diagnosis, like MDQ and BSDS, will be particularly prone to such problems [70]. These instruments can only consider whether BD symptoms are present or absent, never checking or excluding other hypotheses. This increases the probability of random agreement between SDI and NSDI, lowering the estimated reliability (kappa) and also the validity of the diagnosis. Thus, restricting the SDI to a single module likely affects both a tool’s sensitivity and specificity and raises concerns about validity.

Despite having excellent power to evaluate the kappa, we were unable to explain a significant proportion of the heterogeneity in kappa estimates. Heterogeneity was extremely high, and the power to test moderators using a random effects model (as specified a priori) was not optimal. Results are consistent with the possibility that clinicians in “NSDI mode” access different clinical information from SDIs, consequently establishing different diagnoses. Another explanation is that clinicians might be using specific naturalistic and regional prototypes [71] or that diagnostic criteria were interpreted differently across the many cultural contexts. Thus, even if NSDIs and SDIs were targeting the same clinical criteria, there may be differences in how they are framed due to different norms or expectations. Both ICD and DSM manuals draw attention to the possibility that the disorder construct might have relevant differences among people from different countries. Our study included studies from nine different countries on five continents, introducing the possibility of cultural heterogeneity; however, LEI (which differed by country) had no effect in the explanatory model. Finally, linguistic differences may also affect reliability; although SDIs are usually validated after translation, the same could not be said of clinicians using NSDIs.

One initial goal of this study was to examine agreement between SDIs and LEAD standard diagnoses. Despite recent ICD and DSM field trials [40,72], we have not found any paper considering a LEAD gold standard against SDI. Furthermore, the number of codable papers comparing SDI and NSDI diagnoses were bigger than in the Rettew et al. article. Our results show that very few SDIs are actually used. DIGS was not used at all, and most other SDIs have fewer reports when compared with the three most used (SCID, MINI and CIDI). Overall, there is a lack of reporting on the agreement between methods of diagnosis (i.e., LEAD, SDI, NSDI). A major strength of the current work is that it is the only study in the last decade to compare SDIs and NSDIs, a very relevant issue for translational psychiatry. Other relevant strengths were the use of an extraction tool, parallel reviewing strategy, and a very inclusive screening methodology, searching for papers from all continents. It is unlikely that any relevant report was not accessed.

### 4.3. Study Limitations

Due to changes in institutional access, we were unable to screen PsycINFO; although it is unlikely that a relevant journal was indexed in that library, but not in MEDLINE, ISI Web of Science or SCOPUS, that was a departure from our predefined protocol. Additionally, we did not systematically check gray literature and non-indexed journals, which may have resulted in missing smaller studies. However, that would likely have resulted in studies with low kappa, as usually very positive findings are published. This concern is mitigated, however, by the funnel plot we obtained (Figure 5), which points toward a lack of literature with high kappa findings, not low ones.

Working with schizophrenia and BD was a choice as we wanted to measure agreement in two highly valid and prevalent disorders around the world, with supposedly little cultural influence in their definitions across cultures. However, our results cannot be translated to other mental disorders. Indeed, we hypothesize that other disorders might have a poorer reliability performance due to cultural and values interference in NSDI evaluation, which would require further testing outside the scope of this study.

The methodology was not inclusive of comorbidities that might be reasonably prevalent in both disorders. However, failing to diagnose schizophrenia or BD in subjects with other disorders would also be considered an agreement failure, and so we believe that it would have no impact on our findings. Also, our methodology included article types, such as reviews and clinical trials, that would not have been adequately evaluated by our quality tools. The inclusion of these types of articles was a choice in order to increase our sample size, but since none of them were included in the analysis, this methodology option had no impact on the present study.

Finally, the unexplained heterogeneity may jeopardize the interpretation of meta-analysis results. However, the overall estimated kappa aligns with two prior meta-analyses [9,17] as well as what is usually measured in single reports of very well-conducted studies, like Kottwicki [73] longitudinal study of reliability between SDI and NSDI. Moreover, our study used best practices for conducting systematic reviews, including PRISMA guidelines. Thus, since we reached a result that is equivalent to similar studies in the field and employed a rigorous methodology, the heterogeneity warrants consideration as observation in itself rather than as an artifact of our methods.

Reliability has been a major challenge in psychiatry over at least the last 70 years [74]. Most studies showing an increase in reliability with the use of DSM criteria are based on research in academic rather than clinical settings. This reinforces the idea that standardized criteria are not used in clinical practice [11], where a prototype approach may seem more feasible to clinicians [75]. Future work should investigate the extent to which the heterogeneity in agreement between SDIs and NSDIs diagnoses may be attributable to clinicians using clinical prototypes that do not align with categorical diagnostic constructs such as the DSM or to the unreliability of data achieved by SDIs and NSDIs approach.

Our results corroborate previous findings showing only fair kappas between SDIs and NSDIs in clinical settings. Most studies that use SDIs in a previous NSDIs-diagnosed sample do not report the size and results of the tested sample. Also, it is necessary to be more explicit about the full or partial use of an SDI when selecting subjects for research. We would like to suggest that reviewers and journals request this information during the peer review process, but also that guidelines including such information are available for best practices in psychiatry research.

## Figures and Tables

**Table 1:** Inclusion and Exclusion criteria.

Inclusion Criteria	Exclusion Criteria
1. Is it a study that compares agreement between a SDI and a NSDI?	1. Were participants older than 18 and younger than 65 years old? If not, is it possible to separate them from the sample?
2. Is it a study using one of the 11 selected tools? (CIDI, DIS, MINI, SCAN, SCID, SCIP, SADS, DIGD, BSDS, GBI, MDQ, CASH). Refer to which one.	2. Were subjects with intelligence limitation excluded? If not, is it possible to separate them from the sample?
3. Was it published in a peer-reviewed journal?	3. Are SDI and NSDI diagnoses independently obtained?
4. Is it possible to extract diagnostic agreement for schizophrenia or BD? Sign which.	4. Was the NSDI diagnosis obtained by qualified health professional (Physician, Psychiatrist, psychologist or mental health at college grade professional)?
5. Does the reference show kappa agreement between SDI and NSDI? If not, is it possible to calculate it?	5. Was the NSDI diagnosis obtained exclusively for the present study, or was it obtained from medical archive?
	6. Was the diagnosis based on DSM III, DSMIIIr, DSMIV, DSMIVtr, DSMV, ICD 9, ICD 10 or ICD 11?

Footnote: SDI—standard diagnostic interview; NSDI—non-standard diagnostic interview; See Table 2 for SDI acronyms in full length.

**Table 2:** Number of entries by SDI acronym and full-length name.

Standard Diagnostic Interview	Acronym	Full Length
Composite International Diagnostic Interview—CIDI	1162	2662
Diagnostic Interview Schedule—DIS	769	619
Mini International Neuropsychiatric Interview—MINI	10967	2420
Schedules for Clinical Assessment in Neuropsychiatry—SCAN	9259	132
Structured Clinical Interview for DSM—SCID	3103	3872
Standard for Clinicians Interview in Psychiatry—SCIP	88	6
Schedule for Affective Disorders—SADS	584	971
Diagnostic Interview for Genetic Disorders—DIGD	0	0
Bipolar Spectrum Diagnostic Scale—BSDS	35	36
General Behavior Inventory—GBI	42	71
Mood Disorder Questionnaire—MDQ	287	320
The Comprehensive Assessment of Symptoms and History—CASH	1	1
Standard Diagnostic Interview—SDI	31	33

**Table 3:** Selected papers by author, year, diagnosis, sample size, kappa, applied SDI, sample country, clinical scenario, NSDI scenario and SDI applicant.

Author	Year	Diagnosis	Sample Size	Kappa	SDI/Applicant	Country	NSDI Clinical Scenario
Unenge et al. [43]	2012	SCZ	46	-	SCID/Health professional	Sweden	General outpatient
Adelufosi et al. [44]	2012	SCZ	324	-	SCID	Nigeria	General outpatient
Rafrafi et al. [45]	2013	SCZ	114	0.410	CIDI/Health professional	Tunisia	General inpatient
Yazici et al. [46]	2018	SCZ	131	-	SCID	Turkey	Universitary outpatient
Nordgaard et al. [47]	2012	SCZ	100	0.330	SCID/Health professional	Denmark	Universitary inpatient
Stewart et al. [48]	2007	BD	21	-	SCID/Health professional	USA	General inpatient
Zimmerman et al. [49]	2008	BD	700	0.450	SCID/Health professional	USA	Universitary outpatient
Jon et al. [50]	2009	BD	238	0.370	MDQ	South Korea	Universitary outpatient
Vázquez et al. [51]	2010	BD	101	0.550	BSDS	Argentine	General outpatient
Jiménez et al. [52]	2012	BD	138	-	SCID	Spain	Universitary outpatient
Suresh et al. [53]	2013	BD	42	0.250	MDQ	USA	Universitary inpatient
Asaad et al. [54]	2014	BD	390	-	SCID/Health professional	Egypt	General outpatient
Verhoeven et al. [55]	2017	BD	7016	0.480	MINI/Health professional	Netherlands	Universitary outpatient
Ince et al. [56]	2019	BD	183	0.520	BSDS	Turkey	Universitary outpatient
Imamura et al. A [57]	2015	BD	55	0.340	MDQ	Japan	General outpatient
Imamura et al. B [57]	2015	BD	55	0.300	BSDS	Japan	General outpatient
Rajkumar et al. [58]	2016	BD	139	-	MINI	India	General outpatient
Wesley et al. [59]	2018	BD	168	-	MINI/Health professional	India	Universitary outpatient
Hebbrecht et al. [60]	2020	BD	276	0.660	MINI/Health professional	Belgium	Universitary inpatient
Hong et al. [61]	2014	BD	345	0.360	MDQ	South Korea	Universitary outpatient
Lee et al. A [62]	2013	BD	113	0.120	MDQ	South Korea	Universitary inpatient
Lee et al. B [62]	2013	BD	113	0.400	BSDS	South Korea	Universitary inpatient
Kung et al. [63]	2015	BD	860	0.410	MDQ	USA	General inpatient

Footnote: SCZ—schizophrenia; BD—Bipolar Affective Disorder; SCID—Structured Clinical Interview for DSM; CIDI—Composite International Diagnostic Interview; MDQ—Mood Disorder Questionnaire; BSDS—Bipolar Spectrum Diagnostic Scale; MINI—Mini International Neuropsychiatric Interview.

## Data Availability

Not applicable.
